# Draft Genome Sequence of Comamonas testosteroni TA441, a Bacterium That Has a Cryptic Phenol Degradation Gene Cluster

**DOI:** 10.1128/MRA.00946-19

**Published:** 2019-09-26

**Authors:** Hiroyuki Arai, Masaharu Ishii

**Affiliations:** a Department of Biotechnology, Graduate School of Agricultural and Life Sciences, The University of Tokyo, Tokyo, Japan; b Collaborative Research Institute for Innovative Microbiology, The University of Tokyo, Tokyo, Japan; Indiana University, Bloomington

## Abstract

Comamonas testosteroni TA441 has a complete phenol degradation gene cluster but does not degrade phenol because the cluster is tightly repressed. However, mutant strains that can degrade phenol arise by spontaneous mutations of a repressor gene during incubation with phenol. Here, we report the draft genome sequence of strain TA441.

## ANNOUNCEMENT

Comamonas testosteroni strain TA441 was isolated from an enrichment culture of gut homogenates of the wood-feeding termite Reticulitermes speratus, with biphenyl as a carbon source (1). It was selected on an LB plate by having high 2,3-dihydroxybiphenyl dioxygenase (2,3-DHBD) activity. Strain TA441 is not a termite intestinal bacterium because it is an obligate aerobe and has not been identified by the metagenome analysis of the gut microbiota in the termite (2). It is speculated to reside in the soil or wood in the wood-feeding termite ecosystem and utilize lignolytic aromatic compounds as carbon and energy sources (3). The high 2,3-DHBD activity, which catalyzes the aromatic ring cleavage, was found to be involved in the degradation process of steroids, including testosterone and some bile acids, but not in the degradation of biphenyl (4). Strain TA441 has a complete set of the phenol degradation genes but does not grow on phenol as a sole carbon source because the genes are tightly repressed by a repressor, AphS (1, 5, 6). However, mutant strains that can utilize phenol by spontaneous mutations of *aphS* arise after a 2- to 3-week incubation in a medium containing phenol as a carbon source.

Whole-genome sequencing was performed by using paired-end (2 × 300-bp) sequencing on the Illumina MiSeq platform. Total DNA was isolated by a standard phenol-chloroform method from the cells grown in LB medium. DNA fragments at the range of 600 to 720 kb were excised using a LabChip XT (PerkinElmer). DNA fragmentation and sequencing library construction were performed using the HyperPlus kit (Kapa Biosystems), following the instructions from the manufacturer. Adapter sequences and low-quality ends of the row reads were trimmed with FASTX-Toolkit (version 0.0.13) and Sickle (version 1.33). The resultant 2,246,684 read pairs were assembled into 79 contigs of >300 bp with the SPAdes genome assembler (version 3.5.0) with default settings (Illumina). The genome coverage was 236×, and the longest contig length and *N*_50_ size were 924,984 bp and 741,018 bp, respectively. The assembly resulted in a draft genome sequence of 5,707,473 bp with 61.3% GC content. A total of 5,194 protein-coding genes (CDSs) and 90 tRNA genes were detected by the DDBJ Fast Annotation and Submission Tool (DFAST) (https://dfast.nig.ac.jp/).

The genes involved in the degradation of aromatic compounds, such as phenol (1, 5, 6), 3-hydroxybenzoate, and 3-(3-hydroxyphenyl)-propionate/3-hydroxycinnamate (3), and a large gene cluster involved in the degradation of steroids (4, 7) were identified in the genome. Strain TA441 was found to have four terminal oxidases for aerobic respiration. The draft genome sequence of strain TA441 may contribute to elucidation of the adaptation mechanism to the utilization of phenol and will clarify the pathways for degradation of lignolytic compounds and steroids.

### Data availability.

The draft genome sequence of *C. testosteroni* TA441 has been deposited at DDBJ/ENA/GenBank under accession numbers BKBW01000001 to BKBW01000079. The raw sequence data have been deposited at the DDBJ Sequence Read Archive under the accession number DRA008805.
